# Pharmacy practitioners’ attitudes and practice towards herbal products in Jordan: Exploring their knowledge about herbal products potential interactions with cardiovascular medications

**DOI:** 10.12688/f1000research.121709.1

**Published:** 2022-08-08

**Authors:** Rawan Abudalo, Razan Abudalo, Abdelrahim Alqudah, Ayman Abuqamar, Amr Abdelaziz, Maram Alshawabkeh, Luma Taha

**Affiliations:** 1 Department of clinical pharmacy and pharmacy practice, Faculty of Pharmaceutical Sciences, The Hashemite University, Zarqa, Zarqa, 13133, Jordan; 2 Department of Radiology, Jordanian Royal Medical Services, Amman, Jordan; 3 Department of Oncology and Hematology, Jordanian Royal Medical Services, Amman, Jordan

**Keywords:** Herbal products, Pharmacy practitioner, Knowledge, Practice, Herb-cardiovascular drug interaction

## Abstract

**Background:** Herbal medicine use is widespread among patients, as community pharmacies may provide such products. Therefore, pharmacy practitioners should be aware of potential herbal products’ adverse effects and herb-drug interactions, particularly with medications for comorbid diseases, such as cardiovascular drugs, in which pharmacy practitioners need to have good knowledge to provide patients with relevant advice to get optimal and safe therapeutic outcomes. Accordingly, the study is designed to assess the knowledge and awareness of pharmacy practitioners regarding herbal product dispensing and cardiovascular drug interaction in Jordan and view their role in patients’ counselling to set up safe and effective drug use.

**Methods**: A cross-sectional study was conducted in Jordan using an online formatted questionnaire distributed to pharmacy practitioners working in community pharmacies. Descriptive and analytical statistics were performed for the responses using the Statistical Package for the Social Sciences (SPSS) software, version 26.

**Results:** Out of 508 participants, 41.7% had medium knowledge of herbal products pertaining mainly to university education (68.1%); 55.1% of participants dispensed herbal products without prescriptions for obesity and weight reduction (72.8%) and gastrointestinal problems (70.9%); this is because respondents agreed that herbal remedies are safe (28.5%) and effective (38.4%). Whilst the knowledge level of respondents about herbal medicine interaction with cardiovascular medication was medium, with a mean of 1.94, as this interaction may result in potentially serious consequences, 40.7% of respondents strongly agreed to gain more knowledge about the side effects of herbal products and medicine interactions through educational courses.

**Conclusions:** The pharmacy practitioners had medium knowledge of herbal products; however, more attention should be paid to herb-drug interactions in the pharmacy educational curriculum. Additionally, pharmacy practitioners need to refresh their knowledge by attending periodic educational courses and by using reliable resources for information about herbal products in order to provide effective and competent pharmaceutical care.

## Introduction

Herbal medicine consumption has dramatically increased globally. ^1^ This is due to widespread self-medication among populations, as these products are safe with minimum adverse effects, are inexpensive, and have better compatibility and cultural acceptability. ^2^^,^^3^ Additionally, marketing strategies by manufacturers through different methods, such as media, have increased consumers’ awareness about herbal products. ^4^ Nevertheless, the use of herbal medicines, either alone or in combination with other conventional medicines, without informing physicians or pharmacy practitioners may lead to detrimental clinical outcomes.

Herbal product use is common in Arab countries, including Jordan. ^5^ They are available at community pharmacies as registered prepackaged products. Notably, patients use conventional medications concurrently with herbal products for the treatment of various conditions, ^6^ as they are easily purchased without a prescription. However, this has gained significant attention due to the potential risk of drug-herb interactions, as some herbs may mimic, increase, or decrease the action of conventional medications, which is important to consider with drugs that have a narrow therapeutic index, such as warfarin or digoxin. ^7^ Consequently, investigating the safe use of herbal products is considered a major priority.

Herbal product-drug interactions are based on either pharmacokinetic or pharmacodynamic mechanisms. ^8^ Herbal products may alter drug absorption (e.g., pectin reduces lovastatin absorption), ^9^ drug metabolism (e.g., St. John’s wort might increase warfarin metabolism, thus decrease its efficacy), ^10^ or drug renal excretion (e.g., digoxin renal excretion is increased by St. John’s wort). ^11^ Furthermore, such interactions may have an additive or synergistic effect. For example, aspirin’s antiplatelet effect is enhanced by ginkgo biloba. ^12^ On the other hand, some herbal products may antagonize the action of some medications. For example, green tea can antagonize the anticoagulant effect of warfarin as green tea contains small amounts of vitamin K, which may alter warfarin’s action. ^13^

The prevalence of cardiovascular diseases has increased worldwide. ^14^ Most patients have been prescribed anticoagulants, antiplatelets, digoxin, antihypertensives, and antihyperlipidemic agents, which may have a synergistic or antagonistic interaction with herbal products, consequently raising patients’ morbidity and mortality risks. ^10^^,^^15^ Therefore, the use of herbal products should not be initiated without careful consideration of possible drug-herb interactions.

Since pharmacists are the connection between the patient and drug utilization, they are the main attainable health care practitioners ^16^ and they receive more queries about medications. ^17^ Therefore, they play a pivotal role in advising patients and creating awareness of the safe, proper, and rational use of herbal products. ^18^ Nevertheless, to advise patients properly, pharmacists must have noticeable knowledge about herbal products’ therapeutic indications, doses, side effects, and potential interactions with conventional medicines. Inadequate knowledge about herbal products may result in potentially severe adverse events.

Therefore, the present study was conducted to assess the attitude, knowledge, awareness, and dispensing practices among pharmacy practitioners regarding the safe use of herbal drugs and their potential interactions with cardiovascular medications.

## Methods

### Ethical considerations

The study was approved by the Institutional Review Board (IRB) of The Hashemite University, Jordan, with IRB approval code 4/10/2020/2021 that was granted on the 9 ^th^ of May 2021.

### Sample size

According to the Raosoft online calculator (Raosoft), ^19^^,^^20^ and as mentioned by Taherdoost, ^21^ the minimum recommended sample size would be 384 participants at a 95% confidence interval and a 5% margin of error. However, to increase the sample generalizability, a sample size of 508 was collected.

### Study design and data collection

The study was a cross-sectional survey carried out by an anonymous online questionnaire created on Google Forms to obtain the study objectives. The web-based questionnaire was distributed across multiple locations in Jordan through Facebook and WhatsApp applications by the study panel members.

Participants provided a written informed consent statement at the beginning of the questionnaire to decide whether they were willing to participate in the study or not. They were informed that their participation was voluntary and anonymized for researching purposes and was kept confidential to overcome any self-report bias. As well, no specified group was targeted and no leading questions were mentioned in the questionnaire. The sample diversity might add validity and rigor to the obtained results in this study. Eligible participants are pharmacy practitioners of the age of 20 years and older, with either a diploma, bachelor or postgraduates’ education level in pharmacy. Data collection was performed between 11 ^th^ June 2021 till 9 ^th^ September 2021.

The questionnaire was designed to have four domains. The first part consisted of demographic information, such as gender; age, with a minimum of 20 years old; level of education, with at least diploma education; and practicing years. The second part was concerned with pharmacy practitioners’ practice and attitudes towards herbal remedies in pharmacies by choosing any of these responses (always, usually, frequently, and rarely). The third part assessed pharmacy practitioners’ knowledge of the interaction of herbal products, such as ginseng, ginger, gingko biloba, cranberry, and St John’s wort, and cardiovascular medications. Respondents’ level of knowledge was assessed by 3-point Likert scale options as correct, incorrect, or I don’t know answers.

The scale reliability of the interaction of herbs and cardiovascular medications was established by Cronbach’s alpha, which was 0.791; it indicated acceptable levels of reliability with a threshold value of 0.70, as suggested by Hair *et al.,* ^22^ reflecting a high internal consistency among the items.

To determine the minimum and maximum length of a 3-point Likert scale, the range is calculated by (3-1 = 2), then it is divided by three, which is the greatest value of the scale (2 ÷ 3 = 0.66); one is the least value in the scale. Therefore, the scale length is as follows:

• (2.34–3.00) is a high score on the Likert scale, indicating a high level of respondents’ attitudes and perceptions.
• (1.67–2.33) is a medium score on the Likert scale, indicating a medium level of respondents’ attitudes and perceptions.
• (1.00–1.66) is a low score on the Likert scale, indicating a low level of respondents’ attitudes and perceptions.

Finally, the fourth part of the questionnaire assessed pharmacy practitioners’ opinions and beliefs towards herbal products dispensed in community pharmacies using a five-point Likert scale ^23^ with responding options: (1) strongly disagree; (2) disagree; (3) neutral; (4) agree; (5) strongly agree. The scale reliability of respondents’ opinions about herbal products was established by Cronbach’s alpha, which was 0.788; it indicated acceptable levels of reliability with a threshold value of (0.70), as suggested by Hair *et al.,* ^22^ reflecting a high internal consistency among the items. The minimum and maximum length of the 5-point Likert scale is calculated by (5 – 1 = 4), then it is divided by five, which is the greatest value of the scale (4 ÷ 5 = 0.80); one is the least value in the scale. Therefore, the scale length:

• (4.20–5.00) represents a very high score on the Likert scale, indicating a very high level of respondents’ attitudes and perceptions.
• (3.40–4.19) represents a high score on the Likert scale, indicating a high level of respondents’ attitudes and perceptions.
• (2.60–3.39) represents a medium score on the Likert scale, indicating a medium level of respondents’ attitudes and perceptions.
• (1.80–2.59) represents a low score on the Likert scale, indicating a low level of respondents’ attitudes and perceptions.
• (1–1.79) represents a very low score on the Likert scale, indicating a very low level of respondents’ attitudes and perceptions.

However, the final question in part four was about participant’s attitude regarding stopping of conventional medication while using herbal products. Since the questionnaire was simple, it was completed by the respondents without loss of any reading items.

### Statistical analysis

The data were analyzed using the Statistical Package for Social Sciences version 26 (SPSS 26). Percentages and frequencies of responses were calculated for each question. Moreover, mean and standard deviation were calculated for participants’ practice towards herbal products as well as participants’ opinions on the use and dispensing of herbal products in pharmacies to allow for the objective measure of opinion and provide a basis for comparison. Notably, one-way ANOVA F-test analysis was used to identify variables’ relation and dependency by testing the equality of means. A P-value of <0.05 was considered statistically significant.

## Results

### Pharmacy practitioners’ characteristics

A total of 508 pharmacy practitioners from various regions in Jordan responded to the study. ^74^ The demographic responses are shown in Table 1. Based on gender, females responded to the questionnaire more than males (72.2% versus 27.8%). Participants aged between 20 and 29 years old accounted for the highest frequency with 89.8% (456 out of 508). Of these participants, they had varying degrees of education, with the majority having a bachelor’s degree in pharmacy, namely 90.1% (458 out of 508). As well as, they have varying years of experience in practice with 259 of respondents (51%) having 1 to 5 years of experience, while 1.8% participants had more than 20 years of experience. The demographic data are illustrated in Table 1.

**Table 1.** Socio-demographic characteristics of respondents.

Variable	Categorization	Frequency	Percent
Gender	Male	141	27.8
	Female	367	72.2
Age	From 20 – 29 Years	456	89.8
	From 30 – 39 Years	36	7.1
	From 40 – 49 Years	2	0.4
	From 50 – 59 Years	14	2.8
	60 Years and above	-	-
Education level	Diploma	20	3.9
	Bachelor of Pharmacy	458	90.2
	Bachelor's Pharm. D	15	3
	Postgraduate	15	3
Years of experience	Less than 1 Year	15	3
	From 1 – 5 Years	259	51
	From 6 – 10 Years	216	42.5
	From 11 – 15 Years	7	1.4
	From 16 – 20 Years	2	0.4
	Above than 20 Years	9	1.8

### Respondents’ practice and knowledge of herbal products in community pharmacies

According to pharmacy practitioners’ practice towards herbal products, the respondents reported that there is a medium demand for customers to buy herbal products (36.2%), in which 55.1% of respondents said that herbal products are mainly dispensed without prescription due to consumers’ preferences towards self-medications ( Table 2). Notably, the most common herbal products were dispensed for obesity and weight reduction (72.8%) and gastrointestinal disorders (70.9%), whilst 3.7% and 3.1% of herbal products were dispensed for hyperlipidemia and central nervous system disorders respectively, as shown in Table 2.

**Table 2.** Pharmacy practitioners’ practice towards herbal remedies in community pharmacies.

Variable	Categorization	Frequency	Percent
herbal preparations sold more	Prescription	228	44.9
	Without a prescription	280	55.1
Is there a demand for herbal products	Weak	160	30.1
	Medium	184	36.2
	High	164	32.3
Medical conditions for which herbal products were dispensed	Diabetes	211	41.50%
	Hypertension	31	6.10%
	Kidney and urinary system diseases	164	32.30%
	Respiratory problems	40	7.90%
	Digestive problems	360	70.90%
	Central nervous system problems	16	3.10%
	Obesity	370	72.80%
	Hyperlipidaemia	19	3.70%
	Other diseases	133	26.20%

**Figure 1.**
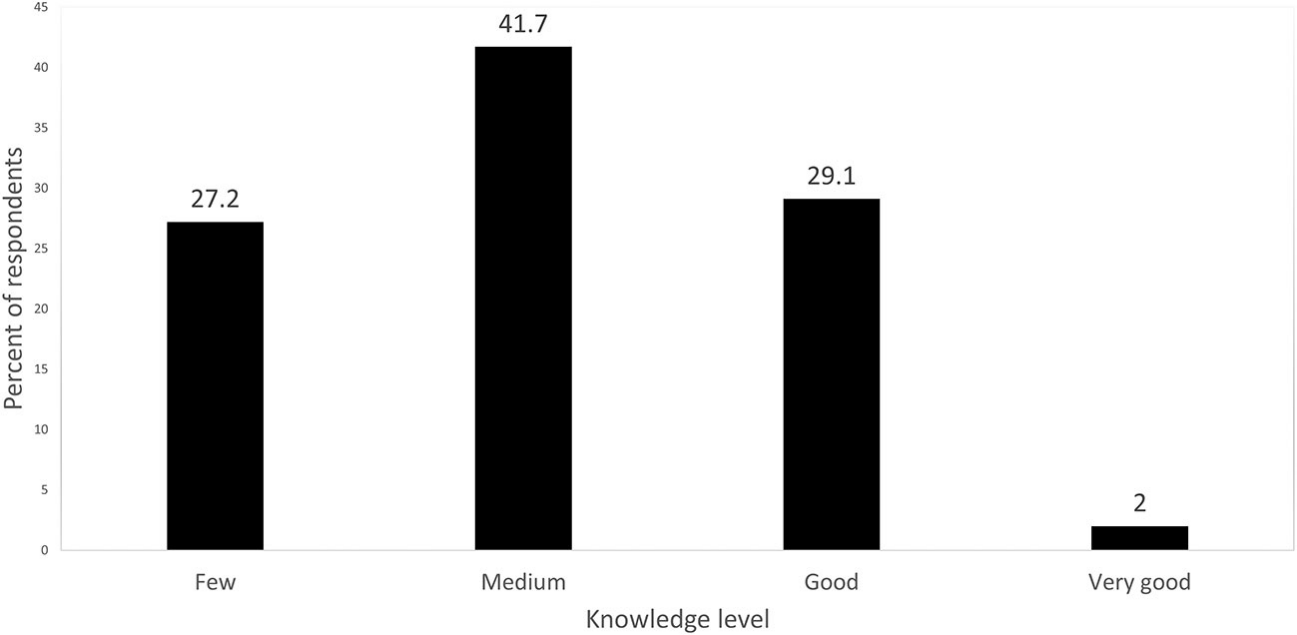
Respondents’ knowledge level regarding herbal products sold in pharmacies.

**Figure 2.**
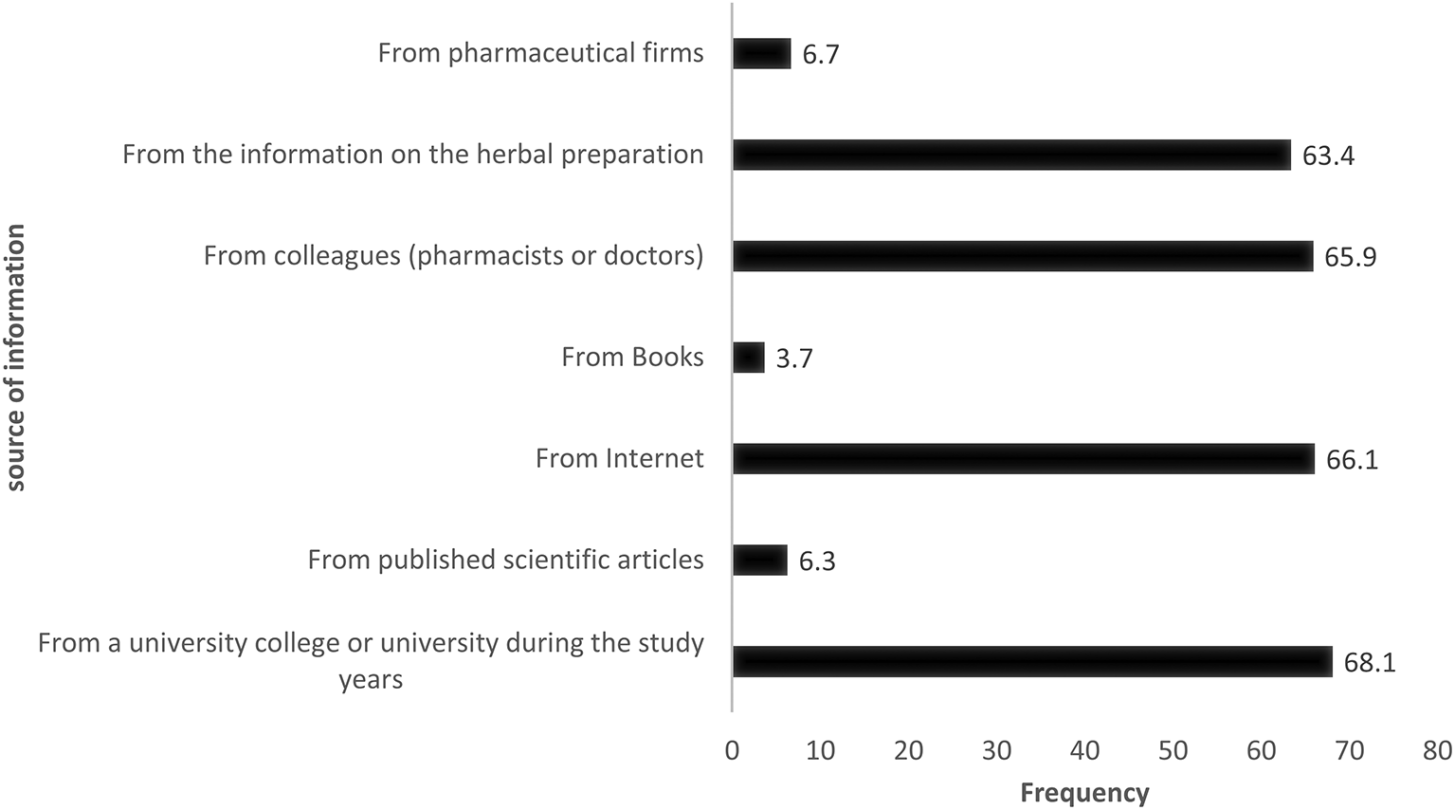
Source of information about herbal products.

When respondents were asked about their knowledge pertaining to herbal products, the majority (41.7%) reported that they had medium knowledge about herbal products in their pharmacies, while only 2% reported that they had very good knowledge ( Figure 1). Regarding the medical and pharmaceutical information they readily had about herbal products, more than two-thirds of the participants (68.1%) relied on university education during their pharmacy study years as the source of knowledge. Other findings showed that 66.1% and 65.9% of pharmacy practitioners relied on websites and their colleagues (pharmacists and physicians) to find the required information about herbal products, respectively. While others mentioned that they obtained their knowledge from herbal products’ package instructions (63.4%) or from pharmaceutical firms’ medical representatives (6.7%), a minority of participants (3.7%) obtained their information from books. The data are shown in Figure 2.

In terms of pharmacy practitioners’ current practicing attitudes towards herbal product dispensing, the results showed that 38.6% of participants usually informed the patient about the condition or medical conditions in which the herbal preparations were used. A total of 35.4% and 35.8% of the participants usually informed the customer about medical conditions that may make herbal preparations unsafe and medications that may interact adversely with herbal preparation, respectively ( Table 3). However, when the respondents were asked about the perceived obstacles in discussing the use of herbal remedies with patients in community pharmacies, the majority (89.4%) answered that there was a lack of sufficient or reliable sources which support the proper and safe use of herbal products, while 82.9% of respondents acknowledged that they had limited interest in herbal product therapy. Likewise, 65% of participants confessed they had inadequate knowledge about herbal products, and this may hinder patient’s herbal products counselling ( Table 4). Therefore, pharmacy practitioners need to be vigilant and knowledgeable about herbal products. This is achieved by providing available herbal medicine resources, as well as by continuing education programs focused on safety and potential herb-drug interactions.

**Table 3.** Pharmacy practitioners’ practicing attitudes towards herbal product dispensing in community pharmacies.

Practicing attitude towards herbal products dispensing	Answer alternatives								Mean	Standard Deviation
	Always		Usually		often		Rarely			
	Freq	%	Freq	%	Freq	%	Freq	%		
The patient has been informed about medical conditions in which the herbal preparations are used	146	28.7	196	38.6	155	30.5	11	2.2	2.939	0.826
The patient has been informed about medical conditions that may make herbal preparations unsafe	172	33.9	180	35.4	133	26.2	23	4.5	2.986	0.885
The patient has been asked about any other medicines he used to avoid drug interactions with herbal preparation	171	33.7	182	35.8	130	25.6	25	4.9	2.982	0.889

**Table 4.** Factors impeding the discussion of herbal medicine use with patients.

Variable	Frequency	Percent
Not caring about the topic	421	82.9
lack of knowledge about herbal products	330	65
lack of sufficient or reliable sources about herbal products and their side effects or drug interaction	454	89.4
Others	19	3.7

### Pharmacy practitioners’ knowledge about herbal products and cardiovascular drug interaction

Regarding pharmacists’ experiences with interactions of herbal products and cardiovascular medications, they were asked 13 questions to evaluate their knowledge level ( Table 5). It was observed that they had a medium level of knowledge (mean = 1.94), with an average of 35.4% of correct answers where they recognized the possible herb and cardiovascular medication interaction, as reported in the literature. The results shown in Table 5 exhibit that an average of 35% of pharmacy practitioners were unaware of the selected herb and cardiovascular drug interaction, and 29.6% didn’t know about the herb-drug interaction.

**Table 5.** Pharmacy practitioners’ knowledge about herbs and cardiovascular drugs interaction.

Herb and cardiovascular drug interaction	Answer alternatives						Mean	Standard Deviation	Level	References
	Correct		Incorrect		I don’t know					
	Freq	%	Freq	%	Freq	%				
Ginkgo biloba can increase the bleeding tendency of warfarin or aspirin	204	40.2	153	30.1	151	29.7	1.90	0.833	Medium	^24^^,^^25^
Ginkgo biloba can decrease the antihypertensive effects of thiazide diureics	147	28.9	200	39.4	161	31.7	2.10	0.821	Medium	^26^
Ginkgo biloba can increase the effectiveness of calcium channel blockers	149	29.3	200	39.4	159	31.3	2.02	0.824	Medium	^10^
Garlic can increase the anticoagulant effect of warfarin	205	40.4	137	27	166	31	1.92	0.810	Medium	^27^
Ginger can increase the risk of bleeding when combined with warfarin	203	40	169	33.3	136	26.8	1.87	0.854	Medium	^28^
Green tea can decrease the anticoagulant action of warfarin	177	34.8	175	34.4	156	30.7	1.96	0.833	Medium	^29^^,^^30^
Evening primrose can increase the risk of bleeding tendency of warfarin, aspirin or clopidogrel	185	36.4	198	39	125	24.6	1.88	0.869	Medium	^31^
St John’s wort may increase blood digoxin level	194	38.2	177	34.8	137	27.0	1.88	0.855	Medium	^32^
Cranberry may increase warfarin anticoagulant effect	168	33.1	186	36.6	154	28.5	1.97	0.835	Medium	^33^
Turmeric can increase the blood level of warfarin and clopidogrel	183	36	180	35.4	145	35.4	1.93	0.846	Medium	^34^
Senna may increase digoxin toxicity	188	37	171	33.7	149	29.3	1.97	0.841	Medium	^15^
Ginseng may decrease warfarin anticoagulant effect	186	36.6	176	34.6	146	28.7	1.92	0.845	Medium	^35^
Coenzyme Q-10 can decrease the effectiveness of warfarin	179	35.2	127	33.9	157	30.9	1.92	0.832	Medium	^36^
Total							1.94	-	**Medium**	

Regarding the association between pharmacy practitioners’ overall knowledge on herbal product-cardiovascular drug interaction and sociodemographic variables, one way ANOVA results established a significant relationship of age (F [3, 504] = 3.161, *p* = 0.024), years of experience (F [5, 502] = 2.594, *p* = 0.025), and educational level (F [3, 504] = 6.674, *p* = 0.000) on pharmacy practitioners overall knowledge levels ( Table 6). Participants aged between 50 to 59 had a higher level of knowledge about herbs and cardiovascular medications interaction than participants aged between 20 to 29 and those aged between 30 to 39 (LSD post hoc, *p* = 0.003, 0.045, respectively). Meanwhile, postgraduate participants scored a higher knowledge level compared to participants with a bachelor’s degree (LSD post hoc, *p* = 0.000). According to work years of experience, pharmacy practitioners who had greater than 20 years of experience had a significantly higher overall knowledge score about herbal product-drug interactions than those who had 1 to 5 and those who had 6 to 10 years of work experience (LSD post hoc, *p* = 0.02, 0.012, respectively). Unexpectedly, participants who had less than one year of experience showed a higher overall knowledge in comparison to those with 6 to 10 years of experience (LSD post hoc, *p* = 0.03).

**Table 6.** Sociodemographic variables’ association to pharmacy practitioners’ overall knowledge on herbal product-cardiovascular drugs interaction.

Variable	Source of variance	Df	Mean square	F	Sig ( *p* value)
**Age**	Between Groups	3	14.853	3.161	0.024
	Within Groups	504	4.699		
	Total	507			
**Education level**	Between Groups	3	30.728	6.674	0.000
	Within Groups	504	4.604		
	Total	507			
**Years of experience**	Between Groups	5	12.152	2.594	0.025
	Within Groups	502	4.685		
	Total	507			

DF, degrees of freedom, *p*, significant value ( *p* < 0.05).

### Pharmacy practitioners’ opinions and beliefs towards herbal products use and dispensing in community pharmacies

When respondents were asked about their opinions on herbal product use and dispensing, level of the use and dispensing of herbal products in pharmacies was high, with mean ranges of 3.69–4.15 and general mean of 3.92. Exactly 38.4% of participants agreed that herbs are effective, with a mean of 3.90 and a standard deviation of 0.804, while 28.5% and 32.3% agreed that herbal products are safe, with fewer adverse effects and with limited drug interactions, respectively. Additionally, 43% of respondents in this study strongly agreed they need to gain more knowledge about herbal products, their side effects and medication interactions, through educational courses and training programs to be highly qualified for better pharmaceutical care services. The results are displayed in Table 7.

**Table 7.** Pharmacy practitioners’ opinions on the use and dispensing of herbal products in pharmacies.

Use and dispensing of herbal products in pharmacies	Answer alternatives										Mean	Standard deviation	Level
	Strongly agree		Agree		Neutral		Disagree		Strongly disagree				
	Freq	%	Freq	%	Freq	%	Freq	%	Freq	%			
Herbal products are effective and able to improve patient's health	133	26.2	195	38.4	175	34.4	4	0.8	1	0.2	3.90	0.804	High
Herbal products are safe and have fewer side effects as they are natural	158	31.1	145	28.5	171	33.7	27	5.3	7	1.4	3.83	0.978	High
Herbal products are safe and have limited drug interactions	143	28.0	164	32.3	156	30.7	38	7.5	7	1.4	3.78	0.983	High
Herbal products are always available at reasonable prices	124	24.4	156	30.7	182	35.8	40	7.9	6	1.2	3.69	0.965	High
The need for medical personnel to gain more knowledge about herbal medicine	219	43.1	151	29.7	136	26.8	-	-	2	0.4	4.15	0.844	High
Importance of providing courses to workers on the use of herbal products, their side effects and medicine interactions	207	40.7	163	32.1	136	26.8	-	-	2	0.4	4.13	0.834	High
Total											3.92	-	High

**Figure 3.**
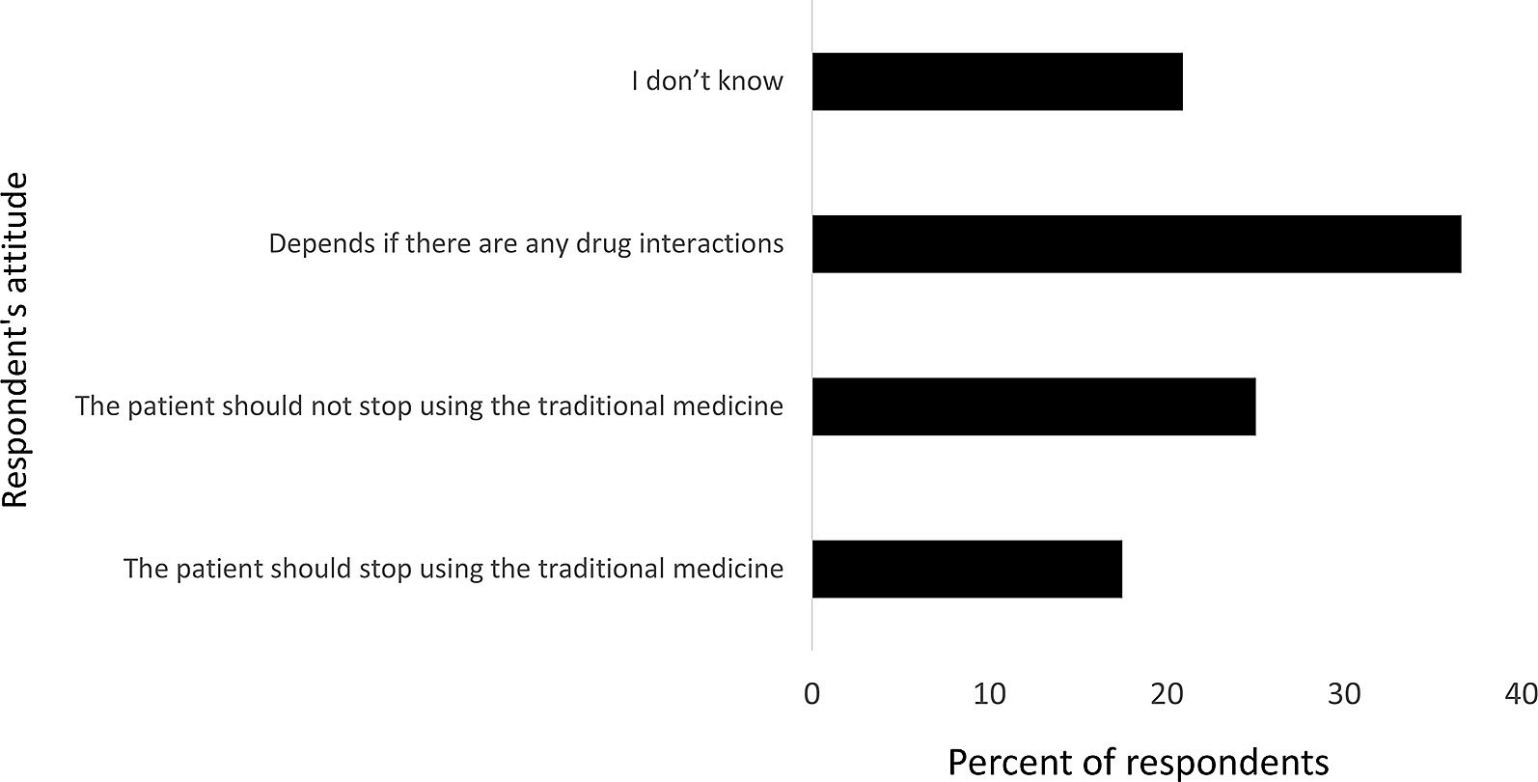
Pharmacy practitioners’ attitude toward stopping the use of conventional medications while using herbal products.

Regarding participants’ attitudes regarding stopping conventional medicine when using herbal products, 36.6% found that it depends on whether there are any drug interactions, 25% believed conventional medication should not be stopped, and 17.5% answered patients should stop traditional medicine. All opinions are summarized in Figure 3.

## Discussion

Herbal product use has increased tremendously globally and in Arab countries, ^37^^,^^38^ including Jordan. ^5^^,^^39^^,^^40^ It has been reported that 80.2% of the laypeople population use herbal products in Jordan ^41^; herbal remedies are favored by individuals, as evidenced by previous studies, because they are natural and, consequently, safe. Meanwhile, herbal products have good efficacy and low cost. ^42^^,^^43^

Knowledge and awareness of drug-herb interactions are substantial for delivering safe and effective drug therapy. Although herbal products are natural and, therefore, safe, ^44^ some herbal products can affect P-glycoprotein drug transporters, cytochrome P450 enzymes, and other metabolic enzymes ^45^; this may result in potential drug interactions, particularly with narrow window medications, if they are taken simultaneously, ^46^ which can result in life-threatening consequences and increase health care costs. Recent studies have demonstrated serious interactions between herbal remedies and medications used in cardiovascular diseases. ^11^^,^^47^^,^^48^ Therefore, this study was the first one conducted in Jordan to assess the knowledge and awareness of pharmacy practitioners concerning the interaction of herbal products and cardiovascular medications.

This study revealed that more than half of respondents dispensed herbal remedies without a prescription, ^49^ specifically for the treatment and control of different illnesses, mainly gastrointestinal problems, ^50^^,^^51^ obesity, ^52^ and diabetes. ^50^ Notably, respondents had medium knowledge about herbal products, which was mainly gained from university during their years of study, where they were exposed to herb-related academic courses in the pharmacy curriculum. ^53^ Other participants chose websites, product leaflets and other colleagues or medical representatives as sources of information about herbal products. ^54^ A large proportion of respondents also relied on the internet to gain knowledge about herbal products; however, media and internet information are not often supervised by a medical or pharmaceutical institution to ensure the accuracy of the information. It has been reported that Missouri’s pharmacists’ knowledge reference towards herbal products was determined by the familiarity of the pharmacists with resource accessibility rather than resource quality. ^55^ Thereby, providing access to authenticated resources, in addition to offering educational programs for pharmacy practitioners about herbal products, is strongly needed.

Despite pharmacy practitioners’ knowledge about herbal products, they usually informed patients about herbal medicine indications, interactions with conventional medications, and conditions that make herbal products unsafe. ^45^ Nevertheless, not caring about herbal products available in community pharmacies impedes pharmacy practitioners from discussing the use of herbal medicines with consumers when purchasing or asking about them, ^56^ this suggests that pharmacy practitioners should be aware about their role in guiding the safe and effective use of herbal products by better communication with patients. ^57^ Additionally, other barriers that could impede pharmacy practitioners and have a considerable influence from discussing herbal remedies are a lack of knowledge or lack of authoritative resources about herbal products. ^58^^,^^59^ To facilitate patients’ acquisition of adequate herbal product counselling, pharmacy practitioners’ education programs and training should be implemented to ascertain the awareness of herbal product counselling and consolidate the provided professional pharmaceutical care services. ^60^^,^^61^ Because the prevalence of cardiovascular diseases has increased dramatically in Jordan, ^62^ this may expand the number of patients’ taking multiple cardiovascular medications in addition to herbal remedies. Some herbal products may have adverse cardiovascular effects, whilst others may interact with cardiovascular medications, such as ginseng, gingko biloba, St. John’s wort, garlic, and induce serious consequences. For example, patients taking anticoagulants, such as warfarin, should avoid the use of ginseng and ginkgo biloba, as they can increase warfarin’s anticoagulant effect, thereby increasing the risk of bleeding. ^63^ Green tea may antagonize warfarin’s anticoagulant effect, ^64^ therefore reducing warfarin’s effectiveness.

St. John’s wort may induce the activity of cytochrome P450 isoenzymes CYP1A2, CYP3A4, and CYP2C9 ^65^; thus, it may reduce the plasma concentration of co-medications like digoxin, ^32^ simvastatin, ^66^ and warfarin, ^67^ which may reduce their therapeutic efficacies. It has been reported that a patient who had used aspirin for five years experienced eye bleeding after the addition of gingko biloba self-medication for one week. ^68^ Therefore, pharmacy practitioners should be vigilant regarding patient counselling on herbal-drug interactions if they are used concurrently with cardiovascular medications to achieve the intended therapeutic outcomes and minimize health complications.

This study revealed that pharmacy practitioners’ overall knowledge score about herbal product interactions with cardiovascular drugs is significantly different based on their age, education level, and years of experience. It was expected that older pharmacy practitioners with more years of work experience and a higher educational level attained, would demonstrate a higher overall knowledge score. Unexpectedly, participants with less than one year of work experience had a significantly higher knowledge score compared to participants who had 6 to 10 years’ experience. The possible explanation is they are newly graduated and have taken courses related to herbal products recently in their studies, as shown in Figure 2, that 68.1% of the respondents relied on their university study courses to gain information about herbal products.

As well, postgraduate education is correlated with more educational courses being studied, which might be relevant to herbal products. However, previous studies have not specifically demonstrated the impact of demographic factors on pharmacy practitioners‘knowledge about herbal product interactions with traditional medications, but they have established that age, education level, and experience (year) influence their general knowledge related to herbal products. ^61^ Regarding the findings of pharmacy practitioners’ practice on the use and dispensing of herbal products, 38.4% agreed that herbal products are effective to improve patient’s health, ^69^ 32.3% agreed they are safe and have limited drug interactions, ^45^ whilst 43.1% strongly agreed they need more knowledge about herbal medicine, which could be achieved by providing courses to workers on the use of herbal products, their side effects, and medicine interactions. Therefore, educational courses on herbal medicine are mandatory. ^70^ However, their attitudes about stopping conventional medication while using herbal products were positive if there were any drug interactions (36.6%), while others said patients should not stop using conventional medicine (25%). ^71^ Patients with chronic diseases who use herbal remedies without informing their physicians, or even physicians who do not guide patients about using these products, may increase potential drug-herb interactions, particularly cardiovascular drugs, which induce detrimental health complications. ^72^ Therefore, a lack of counselling that warns patients about the risk of inappropriate consumption of herbal products will aggravate the condition; pharmacy practitioners play a pivotal role in ensuring patients adhere to the safe use of herbal remedies.

In summary, pharmacy practitioners’ having good knowledge is compulsory for good practice. ^73^ It is essential for pharmacy practitioners to broaden their knowledge pertaining to the adverse effects of herbal products and drug-herb interactions to provide the desired pharmaceutical care to patients, which can be achieved by identifying reliable information resources about herbal products, attending mandatory educational courses on herbal product dispensing in community pharmacies, and advising patients when they are seeking herbal products. All of these recommendations will equip them for good practice, thus, implementing pharmaceutical care services to ascertain the rational and appropriate use of medications.

## Conclusion

The interaction between cardiovascular medications and herbal products is potentially significant, particularly with narrow therapeutic index medications. Because herbal products are more dispensed as over-the-counter medications, and pharmacy practitioners in this study had shown medium knowledge about herb-drug interactions, it is crucial to increase their awareness and knowledge regarding herbal products by educational programs focused on herb indications, drug interactions, and adverse effects. Additionally, it is important for Jordan pharmacists’ association to provide reliable resources. The association plays an important role in collaboration with pharmacy colleges at all Jordanian universities, as pharmacy practitioners in community pharmacies are the main accessible health care providers.

## Consent

Participants provided written informed consent.

## Data availability

### Underlying data

Open Science Framework: Pharmacy Practitioners’ attitudes and practice towards herbal products in Jordan: Exploring their knowledge about herbal products potential interactions with cardiovascular medications. https://doi.org/10.17605/OSF.IO/752DA. ^74^

The project contains the following underlying data:

• Knowledge and awareness of Herbal Products and dietary supplements……xlsx (participants’ responses were coded for SPSS software)
• questionnaire.docx (pharmacy practitioners’ attitude and practice towards herbal products as English version)
• Raw data.sav (participants’ responses to the questionnaire)

Data are available under the terms of the Creative Commons Attribution 4.0 International license (CC-BY 4.0).

## References

[1] Ekor M : The growing use of herbal medicines: issues relating to adverse reactions and challenges in monitoring safety. *Front. Pharmacol.* 2014;4:177. 24454289 10.3389/fphar.2013.00177 PMC3887317

[2] Astin JA : Why patients use alternative medicine: results of a national study. *JAMA.* 1998;279(19):1548–1553. 10.1001/jama.279.19.1548 9605899

[3] Carmona F Pereira AMS : Herbal medicines: old and new concepts, truths and misunderstandings. *Rev. Bras.* 2013;23(2):379–385. 10.1590/S0102-695X2013005000018

[4] Wegener T : Patterns and trends in the use of herbal products, herbal medicine and herbal medicinal products. *Int. J. Complement. Altern. Med.* 2017;9(6):00317. 10.15406/ijcam.2017.09.00317

[5] Issa R Basheti I : Herbal medicine use by people in Jordan: Exploring believes and knowledge of herbalists and their customers. *J. Biol. Sci.* 2017;17(8):400–409. 10.3923/jbs.2017.400.409

[6] Awaisu A : Knowledge, attitudes, and practices of community pharmacists on generic medicines in Qatar. *Int. J. Clin. Pharm.* 2014;36(2):394–404. 10.1007/s11096-013-9909-2 24532363

[7] Izzo A : Drug interactions with St. John's Wort (Hypericum perforatum): a review of the clinical evidence. *Int. J. Clin. Pharmacol. Ther.* 2004;42(3):139–148. 10.5414/CPP42139 15049433

[8] Rosenkranz B Fasinu P Bouic P : An overview of the evidence and mechanisms of herb–drug interactions. *Front. Pharmacol.* 2012;3:69. 22557968 10.3389/fphar.2012.00069 PMC3339338

[9] Richter W Jacob B Schwandt P : Interaction between fibre and Iovastatin. *Lancet.* 1991;338(8768):706. 10.1016/0140-6736(91)91291-2 1679514

[10] Tachjian A Maria V Jahangir A : Use of herbal products and potential interactions in patients with cardiovascular diseases. *J. Am. Coll. Cardiol.* 2010;55(6):515–525. 10.1016/j.jacc.2009.07.074 20152556 PMC2831618

[11] Izzo AA : Cardiovascular pharmacotherapy and herbal medicines: the risk of drug interaction. *Int. J. Cardiol.* 2005;98(1):1–14. 10.1016/j.ijcard.2003.06.039 15676159

[12] Ke J : The Synergistic Effect of Ginkgo biloba Extract 50 and Aspirin Against Platelet Aggregation. *Drug Des. Devel. Ther.* 2021;15:3543–3560. 10.2147/DDDT.S318515 34429584 PMC8375244

[13] Tsai H-H : A Review of Potential Harmful Interactions between Anticoagulant/Antiplatelet Agents and Chinese Herbal Medicines. *PLoS One.* 2013;8(5):e64255. 10.1371/journal.pone.0064255 23671711 PMC3650066

[14] Şahin B İlgün G : Risk factors of deaths related to cardiovascular diseases in World Health Organization (WHO) member countries. *Health Soc. Care Community.* 2022;30(1):73–80. 10.1111/hsc.13156 32909378

[15] Valli G Giardina E-GV : Benefits, adverse effects and drug interactionsof herbal therapies with cardiovascular effects. *J. Am. Coll. Cardiol.* 2002;39(7):1083–1095. 10.1016/S0735-1097(02)01749-7 11923030

[16] Manolakis PG Skelton JB : Pharmacists' Contributions to Primary Care in the United States Collaborating to Address Unmet Patient Care Needs: The Emerging Role for Pharmacists to Address the Shortage of Primary Care Providers. *Am. J. Pharm. Educ.* 2010;74(10):S7. 10.5688/aj7410S7 21436916 PMC3058447

[17] Yang S : A comparison of patients’ and pharmacists’ satisfaction with medication counseling provided by community pharmacies: a cross-sectional survey. *BMC Health Serv. Res.* 2016;16(1):1–8. 10.1186/s12913-016-1374-x 27080704 PMC4832460

[18] Kwan D Hirschkorn K Boon H : US and Canadian pharmacists' attitudes, knowledge, and professional practice behaviors toward dietary supplements: a systematic review. *BMC Complement. Altern. Med.* 2006;6(1):1–10. 16984649 10.1186/1472-6882-6-31 PMC1586212

[19] Raosoft I : Sample size calculator. Reference Source 2004.

[20] McCrum-Gardner E : Sample size and power calculations made simple. *Int. J. Ther. Rehabil.* 2010;17(1):10–14. 10.12968/ijtr.2010.17.1.45988

[21] Taherdoost H : Determining sample size; how to calculate survey sample size. *Int. J. Econ. Manag. Syst.* 2017;2.

[22] Doran RL : *Basic Measurement and Evaluation of Science Instruction.* ERIC;1980.

[23] Vagias WM : Likert-type scale response anchors. *Clemson International Institute for Tourism & Research Development, Department of Parks, Recreation and Tourism Management.* Clemson University;2006.

[24] Jung F : Effect of Ginkgo biloba on fluidity of blood and peripheral microcirculation in volunteers. *Arzneimittelforschung.* 1990;40(5):589–593. 2383302

[25] Skogh M : Extracts of Ginkgo biloba and bleeding or haemorrhage. *Lancet.* 1998;352(9134):1145–1146. 10.1016/S0140-6736(05)79788-9 9798614

[26] Chen X-W : Clinical herbal interactions with conventional drugs: from molecules to maladies. *Curr. Med. Chem.* 2011;18(31):4836–4850. 10.2174/092986711797535317 21919844

[27] Borrelli F Capasso R Izzo AA : Garlic (Allium sativum L.): adverse effects and drug interactions in humans. *Mol. Nutr. Food Res.* 2007;51(11):1386–1397. 10.1002/mnfr.200700072 17918162

[28] Leite PM Martins MAP Castilho RO : Review on mechanisms and interactions in concomitant use of herbs and warfarin therapy. *Biomed. Pharmacother.* 2016;83:14–21. 10.1016/j.biopha.2016.06.012 27470545

[29] Green GA Catlin DH Starcevic B : Analysis of over-the-counter dietary supplements. *Clin. J. Sport Med.* 2001;11(4):254–259. 10.1097/00042752-200110000-00008 11753063

[30] Taylor JR Wilt VM : Probable antagonism of warfarin by green tea. *Ann. Pharmacother.* 1999;33(4):426–428. 10.1345/aph.18238 10332534

[31] Fugh-Berman A : Herb-drug interactions. *Lancet.* 2000;355(9198):134–138. 10.1016/S0140-6736(99)06457-0 10675182

[32] Johne A : Pharmacokinetic interaction of digoxin with an herbal extract from St John's wort (Hypericum perforatum). *Clin. Pharmacol. Ther.* 1999;66(4):338–345. 10.1053/cp.1999.v66.a101944 10546917

[33] Hamann GL Campbell JD George CM : Warfarin-cranberry juice interaction. *Ann. Pharmacother.* 2011;45(3):e17. 10.1345/aph.1P451 21364039

[34] Wang Z-Y : Pharmacokinetic drug interactions with clopidogrel: updated review and risk management in combination therapy. *Ther. Clin. Risk Manag.* 2015;11:449. 25848291 10.2147/TCRM.S80437 PMC4373598

[35] Janetzky K Morreale AP : Probable interaction between warfarin and ginseng. *Am. J. Health Syst. Pharm.* 1997;54(6):692–693. 10.1093/ajhp/54.6.692 9075501

[36] Bonakdar RA Guarneri E : Coenzyme Q10. *Am. Fam. Physician.* 2005;72(6):1065–1070. 16190504

[37] AlBraik FA Rutter PM Brown D : A cross-sectional survey of herbal remedy taking by United Arab Emirate (UAE) citizens in Abu Dhabi. *Pharmacoepidemiol. Drug Saf.* 2008;17(7):725–732. 10.1002/pds.1591 18395880

[38] Alsayari A : Community Pharmacists' Knowledge, Attitudes, and Practice of Herbal Medicines in Asir Region, Kingdom of Saudi Arabia. *Evid. Based Complement. Alternat. Med.* 2018;2018:1568139. 30228824 10.1155/2018/1568139 PMC6136488

[39] Khader Y : Knowledge and attitudes of lay public, pharmacists, and physicians toward the use of herbal products in North Jordan. *J. Altern. Complement. Med.* 2008;14(10):1186–1187. 10.1089/acm.2008.0282 19040389

[40] Issa R Basheti I : Herbal products use among chronic patients and its impact on treatments safety and efficacy: a clinical survey in the Jordanian field. *Trends Med. Res.* 2017;12:32. 2016. 44.

[41] El-Dahiyat F : Herbal medicines: a cross-sectional study to evaluate the prevalence and predictors of use among Jordanian adults. *J. Pharm. Policy Pract.* 2020;13:2. 10.1186/s40545-019-0200-3 31988754 PMC6971905

[42] Cock I : The safe usage of herbal medicines: counter-indications, cross-reactivity and toxicity. *Pharmacognosy Communications.* 2015;5(1).

[43] Joos S Glassen K Musselmann B : Herbal medicine in primary healthcare in Germany: the Patient's perspective. *Evid. Based Complement. Alternat. Med.* 2012;2012:1–10. 10.1155/2012/294638 23346197 PMC3549419

[44] AlBraik FA Rutter PM Brown D : A cross-sectional survey of herbal remedy taking by United Arab Emirate (UAE) citizens in Abu Dhabi. *Pharmacoepidemiol. Drug Saf.* 2008;17(7):725–732. 10.1002/pds.1591 18395880

[45] Carr A Santanello C : Pharmacists’ Knowledge, Perceptions, and Practices Regarding Herbal Medicine. *Innov. Pharm.* 2019;10(3). 10.24926/iip.v10i3.2059 PMC8127100 34007576

[46] Alissa EM : Medicinal herbs and therapeutic drugs interactions. *Ther. Drug Monit.* 2014;36(4):413–422. 10.1097/FTD.0000000000000035 24452064

[47] Cohen PA Ernst E : Safety of herbal supplements: a guide for cardiologists. *Cardiovasc. Ther.* 2010;28(4):246–253. 10.1111/j.1755-5922.2010.00193.x 20633025

[48] Tachjian A Maria V Jahangir A : Use of herbal products and potential interactions in patients with cardiovascular diseases. *J. Am. Coll. Cardiol.* 2010;55(6):515–525. 10.1016/j.jacc.2009.07.074 20152556 PMC2831618

[49] Abdel-Qader DH : Herbal medicines use in the Jordanian population: A nationally representative cross-sectional survey. *J. Pharm. Pharmacogn. Res.* 2020;8(2).

[50] Younis NAKY Prevalence T : Attitude and Awareness of Herbal Medicine Products Use Among Pharmacy Practitioner in Jordan. *Pharmacogn. J.* 2019;11(5):1082–1087. 10.5530/pj.2019.11.169

[51] Darwish RM : Users’ Knowledge and Self Medications in Relation to Gastric Problems among Adults in a Middle Income Country; Jordan. *J. Subst. Abus.* 2021:1–5. 10.1080/14659891.2021.1961323

[52] Al-Safi SA : Public awareness of the abuse of herbs and drugs to decrease body weight: a novel national survey in Jordan. *J. Public Health.* 2008;16(3):205–213. 10.1007/s10389-007-0166-5

[53] Abdelmalek S Alkhawaja B Darwish D : Perceptions and use of medicinal herbs among college students at a Jordanian University in Amman-Jordan: traditions supersedes education. *J. Tradi. Med. Clin. Natur.* 2016;5(191):2.

[54] Khdour MR : Pharmacists' Knowledge, Attitudes and Practices Towards Herbal Remedies In West Bank, Palestine. *Int. Arch. Med.* 2016;9. 10.3823/1974

[55] Clauson KA : Knowledge and attitudes of pharmacists in Missouri regarding natural products. *Am. J. Pharm. Educ.* 2003;67(1/4):301.

[56] Bouwmeester CJ : Surveying physicians' attitudes about herbal supplements, resources, and pharmacy consultations. *J. Pharm. Technol.* 2005;21(5):247–253. 10.1177/875512250502100502

[57] Lin H-W : An instrument to evaluate pharmacists' patient counseling on herbal and dietary supplements. *Am. J. Pharm. Educ.* 2010;74(10):192. 10.5688/aj7410192 21436933 PMC3058472

[58] Al-Arifi MN : Availability and needs of herbal medicinal information resources at community pharmacy, Riyadh region, Saudi Arabia. *Saudi Pharm. J.* 2013;21(4):351–360. 10.1016/j.jsps.2012.11.004 24227954 PMC3824944

[59] Bhadra R Ravakhah K Ghosh RK : Herb-drug interaction: The importance of communicating with primary care physicians. *Australas Med. J.* 2015;8(10):315–319. 10.4066/AMJ.2015.2479 26576202 PMC4643608

[60] Basheti IA : Opinions of pharmacists and herbalists on herbal medicine use and receiving herbal medicine education in Jordan. *Trop. J. Pharm. Res.* 2017;16(3):689–696. 10.4314/tjpr.v16i3.26

[61] Aiesh BM : Community Pharmacists’ Patterns of Use, Knowledge, and Attitudes toward Complementary and Alternative Medicine (CAM) in Palestine: A cross-sectional Study. 2015.

[62] Alsaud W : Prevalence of cardiovascular Diseases Risk Factors Among Jordanians. *J. Saudi Heart Assoc.* 2020;32(2):324–333. 10.37616/2212-5043.1074 33154938 PMC7640553

[63] Ge B Zhang Z Zuo Z : Updates on the clinical evidenced herb-warfarin interactions. *Evid. Based Complement. Alternat. Med.* 2014;2014:1–18. 10.1155/2014/957362 24790635 PMC3976951

[64] Lam AY Elmer GW Mohutsky MA : Possible interaction between warfarin and *Lycium barbarum* L. *Ann. Pharmacother.* 2001;35(10):1199–1201. 10.1345/aph.1Z442 11675844

[65] Mannel M : Drug interactions with St John’s wort. *Drug Saf.* 2004;27(11):773–797. 10.2165/00002018-200427110-00003 15350151

[66] Sugimoto K : Different effects of St John's wort on the pharmacokinetics of simvastatin and pravastatin. *Clin. Pharm. Ther.* 2001;70(6):518–524. 10.1067/mcp.2001.120025 11753267

[67] Henderson L : St John's wort (Hypericum perforatum): drug interactions and clinical outcomes. *Br. J. Clin. Pharmacol.* 2002;54(4):349–356. 10.1046/j.1365-2125.2002.01683.x 12392581 PMC1874438

[68] Rosenblatt M Mindel J : Spontaneous hyphema associated with ingestion of Ginkgo biloba extract. *N. Engl. J. Med.* 1997;336(15):1108–1108. 10.1056/NEJM199704103361518 9091822

[69] Calixto J : Efficacy, safety, quality control, marketing and regulatory guidelines for herbal medicines (phytotherapeutic agents). *Braz. J. Med. Biol. Res.* 2000;33(2):179–189. 10.1590/S0100-879X2000000200004 10657057

[70] Saad B : Safety of traditional Arab herbal medicine. *Evid. Based Complement. Alternat. Med.* 2006;3(4):433–439. 10.1093/ecam/nel058 17173106 PMC1697757

[71] Abahussain NA Abahussain EA Al-Oumi FM : Pharmacists' attitudes and awareness towards the use and safety of herbs in Kuwait. *Pharm. Pract. (Internet).* 2007;5(3):125–129. 10.4321/S1886-36552007000300005 PMC4154746 25214928

[72] Kelak JA Cheah WL Safii R : Patient’s decision to disclose the use of traditional and complementary medicine to medical doctor: a descriptive phenomenology study. *Evid. Based Complement. Alternat. Med.* 2018;2018:1–11. 10.1155/2018/4735234 29636778 PMC5832099

[73] Hanafi S : Evaluation of community pharmacists’ knowledge, attitude and practice towards good pharmacy practice in Iran. *J. Pharm. Care.* 2013:19–24.

[74] Alqudah A : Pharmacy Practitioners’ attitudes and practice towards herbalproducts in Jordan: Exploring their knowledge about herbal products potential interactions with cardiovascular medications. *Open Science Framework (OSF.)* 23 May 2022. 10.17605/OSF.IO/752DA

