# Supplementary material for: Pharmacy practitioners’ practice, awareness and knowledge about herbal products and their potential interactions with cardiovascular drugs
Source: F1000Res. 2022 Sep 22;11:912. Originally published 2022 Aug 8. [Version 2] doi: 10.12688/f1000research.121709.2 (PMC9520230; doi:10.12688/f1000research.121709.2)
Supplement: Peer review history

# Peer review: Reviewer response for version 2

**DOI:** 10.5256/f1000research.138508.r151163

**Published:** 2022-09-28

**Authors:** Banaz Jalil

**Affiliations:** 1 Pharmacognosy and Phytotherapy, UCL School of Pharmacy, London, UK

Thanks for the revised manuscript! The authors have addressed the reviewer's comments in their response and manuscript revisions. Therefore, I have no further comments.

Is the work clearly and accurately presented and does it cite the current literature?

Yes

If applicable, is the statistical analysis and its interpretation appropriate?

Yes

Are all the source data underlying the results available to ensure full reproducibility?

Yes

Is the study design appropriate and is the work technically sound?

Yes

Are the conclusions drawn adequately supported by the results?

Yes

Are sufficient details of methods and analysis provided to allow replication by others?

Yes

Reviewer Expertise:

Social and Pharmaceutical Sciences, Medicinal Plants Research, Pharmacy Practice and Research Policy.

I confirm that I have read this submission and believe that I have an appropriate level of expertise to confirm that it is of an acceptable scientific standard.

# Peer review: Reviewer response for version 2

**DOI:** 10.5256/f1000research.138508.r151162

**Published:** 2022-09-23

**Authors:** Muna Barakat

**Affiliations:** 1 Faculty of Pharmacy, Applied Science Private University, Amman, Jordan

Thank you for addressing the comments. From my side, it looks better and is suitable for indexing in its current form.

Is the work clearly and accurately presented and does it cite the current literature?

Yes

If applicable, is the statistical analysis and its interpretation appropriate?

Yes

Are all the source data underlying the results available to ensure full reproducibility?

Yes

Is the study design appropriate and is the work technically sound?

Yes

Are the conclusions drawn adequately supported by the results?

Yes

Are sufficient details of methods and analysis provided to allow replication by others?

Yes

Reviewer Expertise:

Pharmacy Practice and clinical pharmacy

I confirm that I have read this submission and believe that I have an appropriate level of expertise to confirm that it is of an acceptable scientific standard.

# Peer review: Reviewer response for version 1

**DOI:** 10.5256/f1000research.133604.r146934

**Published:** 2022-09-01

**Authors:** Banaz Jalil

**Affiliations:** 1 Pharmacognosy and Phytotherapy, UCL School of Pharmacy, London, UK

Thanks for the review invitation. The authors surveyed pharmacists' knowledge of herbal products and their awareness of potential interactions with conventional medicines in Jordan (e.g., cardiovascular medications). An online questionnaire was used in this study. In total, 508 participants have completed the study. The overall knowledge scores about herbal product interactions with cardiovascular medications differed based on the participant's age, education level, and years of experience. I believe the following areas could be strengthened before indexing:

1. The title is wordy, so it would be good to revise it.
2. Please use consistent terms to refer to herbal medicines, herbal products and/or herbs throughout the manuscript, preferably reflecting the terms used in the survey. The same goes for the terms ‘medications and drugs’. Also, 'Participants and Respondents'.
3. Instead of using the term ‘herbal product-drug interaction’, please use ‘herb-drug interactions throughout the manuscript.
4. Please add a paragraph on how the survey tool was developed, was it based on previous studies or developed by the authors? Please cite other studies if pre-validated questionnaires were used. If developed by the authors, then how was it validated?
5. Table 2, it will be better if you list the results (either by frequency or %) from high to low.
6. In the ‘Respondents’ practice and knowledge of herbal products in community pharmacies’ section, it’s better to use two-thirds, a quarter, half etc., to refer to the participants' responses rather than the majority and/or minority.
7. Finally, although the manuscript is clear and well written, editing is needed, especially for the introduction, method, and results sections. There is also some repetition in these areas. So, I would suggest proofreading the manuscript.

Is the work clearly and accurately presented and does it cite the current literature?

Yes

If applicable, is the statistical analysis and its interpretation appropriate?

Yes

Are all the source data underlying the results available to ensure full reproducibility?

Yes

Is the study design appropriate and is the work technically sound?

Yes

Are the conclusions drawn adequately supported by the results?

Yes

Are sufficient details of methods and analysis provided to allow replication by others?

Yes

Reviewer Expertise:

Social and Pharmaceutical Sciences, Medicinal Plants Research and Pharmacy Practice.

I confirm that I have read this submission and believe that I have an appropriate level of expertise to confirm that it is of an acceptable scientific standard, however I have significant reservations, as outlined above.

# Response

**Published:** 2022-09-10

**Authors:** Abdelrahim qudhah

We would like to thank you for the invested time and effort in carefully reviewing our manuscript. We are grateful for giving us the opportunity to revise our manuscript. Your comments were very useful and helped us in improving our manuscript. After careful consideration of the comments, the revision included many positive changes as suggested.

 C1: The title is wordy, so it would be good to revise it.

 R1: Thank you for your comment. Title was amended as follows: ‘’Pharmacy practitioners’ Practice, awareness and knowledge about herbal products and their potential interactions with cardiovascular drugs.’’

 C2: Please use consistent terms to refer to herbal medicines, herbal products and/or herbs

 throughout the manuscript, preferably reflecting the terms used in the survey. The same

 goes for the terms ‘medications and drugs’. Also, 'Participants and Respondents

 R 2: Thank you for your comment. Terms were amended as:

- Herbal medicines term was replaced by herbal products.
- Medications term was replaced by drugs.
- Participants term was replaced by Respondents

C 3: Instead of using the term ‘herbal product-drug interaction’, please use ‘herb-drug interactions throughout the manuscript.

 R 3: Thank you for your comment. ‘herbal product-drug interaction’ was amended as ‘herb-drug interactions.

 C 4: Please add a paragraph on how the survey tool was developed, was it based on previous

 studies or developed by the authors? Please cite other studies if pre-validated questionnaires were used. If developed by the authors, then how was it validated?

 R4: Thank you for your comment Kindly find this paragraph. A structured questionnaire was created for the purpose of this study, and was validated by a group of experts constituted of two pharmacologists, Phytochemist, cardiologist, and pharmacist. 

 Piloting of the questionnaire was performed to assess the accuracy and comprehension of the questions in relation to the research topic, and to identify possible redundancy. The knowledge scale as well as opinions and beliefs towards herbal products scale were calculated and showed an excellent reliability with a Cronbach’s alpha coefficient of 0.791, and 0.788 respectively indicating a high internal consistency among the items. However, responses completed during the piloting phase were excluded from the final analysis.

 The knowledge scale was assessed for validity by examining the content validity index (CVI). Five experts were consulted for their opinion for each scale item including clarity, simplicity and relevancy and scores from 1 (strongly disagreed), 2 (disagreed), 3 (agreed) and 4 (strongly agreed). Questions were tested/re-tested for their clarity and simplicity by interviewing 20 participants. The CVI were ranged from 0.8-0.9 and it was deemed that the scale was valid. 

 C5: Table 2, it will be better if you list the results (either by frequency or %) from high to low.

 R5: Thank you for your comment, table 2 has been amended.

 C6: In the ‘Respondents’ practice and knowledge of herbal products in community pharmacies’ section, it’s better to use two-thirds, a quarter, half etc., to refer to the participants'

 responses rather than the majority and/or minority.

 R6: Thank you for your comment, Words have been amended as:

 ‘Majority is replaced by more than third, minority is replaced by lowest percent of respondents.

 C7: Finally, although the manuscript is clear and well written, editing is needed, especially for the introduction, method, and results sections. There is also some repetition in these areas. So, I would suggest proofreading the manuscript.

 R7: Thank you for your comment. The manuscript is now proofread by Ryan Gibson Proofreading Services in UK.

# Peer review: Reviewer response for version 1

**DOI:** 10.5256/f1000research.133604.r146933

**Published:** 2022-08-18

**Authors:** Muna Barakat

**Affiliations:** 1 Faculty of Pharmacy, Applied Science Private University, Amman, Jordan

Thank you for inviting me to review this work, "Pharmacy practitioners’ attitudes and practice towards herbal products in Jordan: Exploring their knowledge about herbal products potential interactions with cardiovascular medications". This manuscript discusses a very important research area; hence, Jordanians believe herbal medicine is a safe replacement for chemical medications. So, it is worth it to investigate the KAP of the practitioners towards herbs.

 I believe this work is suitable for indexing after addressing the comments:

Title:

- In my opinion, the title could be rephrased comprehensively, for example, "Pharmacists' Knowledge, Practice and awareness about herbal products and their potential interactions with cardiovascular medications"

Introduction:

- I believe you need to add a small paragraph about Jordan's context, and clarify the research gap. 

Methods:

- Add the inclusion criteria, please.

Statistical analysis:

- You need to add if you have checked the data normality and the used test.

Results:

- Figure 2, no need to add "From" before each source of information and reorder them according to percentages. 
- Table 3. I believe it is important to calculate the overall mean of attitude, to express the level of attitude concisely. 

Is the work clearly and accurately presented and does it cite the current literature?

Yes

If applicable, is the statistical analysis and its interpretation appropriate?

Yes

Are all the source data underlying the results available to ensure full reproducibility?

Yes

Is the study design appropriate and is the work technically sound?

Yes

Are the conclusions drawn adequately supported by the results?

Yes

Are sufficient details of methods and analysis provided to allow replication by others?

Yes

Reviewer Expertise:

Pharmacy Practice and clinical pharmacy

I confirm that I have read this submission and believe that I have an appropriate level of expertise to confirm that it is of an acceptable scientific standard.

# Response

**Published:** 2022-09-10

**Authors:** Abdelrahim qudhah

We would like to thank you for the invested time and effort in carefully reviewing our manuscript. We are grateful for giving us the opportunity to revise our manuscript. Your comments were very useful and helped us in improving our manuscript. After careful consideration of the comments, the revision included many positive changes as suggested.

 C1: In my opinion, the title could be rephrased comprehensively, for example, "Pharmacists'

 Knowledge, Practice and awareness about herbal products and their potential interactions

 with cardiovascular medications

 R1: Thank you for your comment. Title was amended as follows: ‘’Pharmacy practitioners’ Practice, awareness and knowledge about herbal products and their potential interactions with cardiovascular drugs.’’

 C2: Introduction: I believe you need to add a small paragraph about Jordan's context, and clarify the research gap.

 R2: Thank you for your comment. Kindly find this paragraph:

 Several studies have been conducted in Arab countries assess pharmacists’ knowledge and attitudes towards herbal products[1-4]. In Jordan, the prevalence and awareness of herbal products used among pharmacy staff have been measured which revealed that pharmacists’ awareness about herbal products indication was good. However, their knowledge of herb’s adverse effects, and drug interactions were inadequate[5] which sounded alarm. Additionally, no studies focused on Jordanian pharmacy practitioners’ knowledge and awareness towards herb-drug interactions particularly with cardiovascular drugs as they are commonly used in Jordan. Thereby, the study’s investigators strive to increase the attention of pharmacy practitioners regarding herb-drug interaction that may lead to toxic manifestations. Likewise, investigators seek to reinforce the need of periodic education programs for pharmacy practitioners focused on herbal products indications, side effects and drug interactions to refresh their knowledge for competent and holistic and health care as this has been documented by other study conducted in Jordan [6].

 C3: Methods-Add the inclusion criteria, please.

 R3: Thank you for your comment, we included the pharmacy practitioners in community pharmacies and excluded those who working in other places.

 C4: Statistical analysis: You need to add if you have checked the data normality and the used test?

 R4: Thank you for your comment, the data normality was checked using the Kolmogorov–Smirnov test.

 C5: Results: Figure 2, no need to add "From" before each source of information and reorder them according to percentages.

 R5: Thank you for your comment, figure 2 has been amended.

 C6: Table 3. I believe it is important to calculate the overall mean of attitude, to express the level of attitude concisely.

 R6: Thank you for your comment, you are right. However, the mean values of the measured variables are very close, thereby it is uncapable to setup a range and to express the overall level of attitude is high, medium or low.
